# Function of Succinoglycan Polysaccharide in *Sinorhizobium meliloti* Host Plant Invasion Depends on Succinylation, Not Molecular Weight

**DOI:** 10.1128/mBio.00606-16

**Published:** 2016-06-21

**Authors:** Hajeewaka C. Mendis, Thelma F. Madzima, Clothilde Queiroux, Kathryn M. Jones

**Affiliations:** Department of Biological Science, Florida State University, Tallahassee, Florida, USA

## Abstract

The acidic polysaccharide succinoglycan produced by the rhizobial symbiont *Sinorhizobium meliloti* 1021 is required for this bacterium to invade the host plant *Medicago truncatula* and establish a nitrogen-fixing symbiosis. *S. meliloti* mutants that cannot make succinoglycan cannot initiate invasion structures called infection threads in plant root hairs. *S. meliloti* exoH mutants that cannot succinylate succinoglycan are also unable to form infection threads, despite the fact that they make large quantities of succinoglycan. Succinoglycan produced by *exoH* mutants is refractory to cleavage by the glycanases encoded by *exoK* and *exsH*, and thus succinoglycan produced by *exoH* mutants is made only in the high-molecular-weight (HMW) form. One interpretation of the symbiotic defect of *exoH* mutants is that the low-molecular-weight (LMW) form of succinoglycan is required for infection thread formation. However, our data demonstrate that production of the HMW form of succinoglycan by *S. meliloti* 1021 is sufficient for invasion of the host *M. truncatula* and that the LMW form is not required. Here, we show that *S. meliloti* strains deficient in the *exoK*- and *exsH-*encoded glycanases invade *M. truncatula* and form a productive symbiosis, although they do this with somewhat less efficiency than the wild type. We have also characterized the polysaccharides produced by these double glycanase mutants and determined that they consist of only HMW succinoglycan and no detectable LMW succinoglycan. This demonstrates that LMW succinoglycan is not required for host invasion. These results suggest succinoglycan function is not dependent upon the presence of a small, readily diffusible form.

## IMPORTANCE

*Sinorhizobium meliloti* is a bacterium that forms a beneficial symbiosis with legume host plants. *S. meliloti* and other rhizobia convert atmospheric nitrogen to ammonia, a nutrient source for the host plant. To establish the symbiosis, rhizobia must invade plant roots, supplying the proper signals to prevent a plant immune response during invasion. A polysaccharide, succinoglycan, produced by *S. meliloti* is required for successful invasion. Here, we show that the critical feature of succinoglycan that allows infection to proceed is the attachment of a “succinyl” chemical group and that the chain length of succinoglycan is much less important for its function. We also show that none of the short-chain versions of succinoglycan is produced in the absence of two chain-cleaving enzymes.

## INTRODUCTION

*Sinorhizobium meliloti* 1021 is a soil bacterium and nitrogen-fixing symbiont of the host plants *Medicago truncatula* cv. “Jemalong A17” and *Medicago sativa* (alfalfa) (1, 2). Under nitrogen-limiting conditions, *S. meliloti* induces formation of nodules on host plant roots, invades and colonizes the nodules (1, 2), and then begins to convert or “fix” dinitrogen gas to ammonia, a form that the host can use (2). For successful invasion of host plant roots by rhizobia, the symbiotic partners must exchange multiple signals that promote bacterial entry. Plant flavonoids signal *S. meliloti* to produce a lipochitooligosaccharide signal called Nod factor (NF) (3). NF induces host plant root hair curling that leads to trapping of microcolonies of *S. meliloti* within the curl and induces cell division in the root cortex, leading to formation of the nodule primordium (2). Structures called infection threads initiate from these colonized curled root hairs. An infection thread is a progressive ingrowth of root hair cell membrane that leaves behind a tubule filled with *S. meliloti* and a matrix composed of bacterial exopolysaccharides (EPS) and plant cell wall material (4, 5). It is through infection threads that rhizobia invade and colonize the root interior (1). Infection thread initiation and development require that *S. meliloti* propagate in the infection thread and produce both NF and the EPS succinoglycan (1). Infection threads in root hairs are extended to the base of these cells and through each cell layer, eventually delivering the bacteria to proliferating cells of the nodule primordium (6, 7). Succinoglycan production by *S. meliloti* 1021 is required for this bacterium to induce infection threads on host plants (8). *S. meliloti* 1021 strains that do not produce succinoglycan, such as the *exoY* mutant (9), are able to colonize root surfaces and become tightly enclosed within curled root hairs but fail to initiate infection threads (10).

Rhizobial acidic EPSs are either required for or enhance host invasion in multiple rhizobial symbiont-host plant pairs (11–25). In some cases, determining the importance of a symbiotic EPS in host invasion has been complicated by production of multiple EPSs by a single bacterial strain. However, in the *S. meliloti* 1021-*M. truncatula* host-symbiont pair, succinoglycan is the only EPS produced in sufficient quantities and in a functional form that can enable infection thread formation (8, 19, 21, 23, 26) (see Discussion). It has also been demonstrated that increased succinoglycan production by *S. meliloti* leads to an increase in symbiotic productivity of inoculated *M. truncatula* plants (27). Acidic EPSs of bacterial pathogens of plants can also be virulence determinants in plant disease. Many of these negatively charged polysaccharides have been shown to suppress plant defense activation by sequestering Ca^2+^ and preventing a signaling cascade (28). Both the EPS xanthan of *Xanthomonas campestris* pv. *campestris* (29, 30) and the EPS alginate of *Pseudomonas syringae* (31, 32) enhance host plant infection by these pathogens and exacerbate disease symptoms. It is not yet known if acidic EPSs of rhizobial plant symbionts and of plant pathogens perform any similar functions in host invasion.

**FIG 1:**
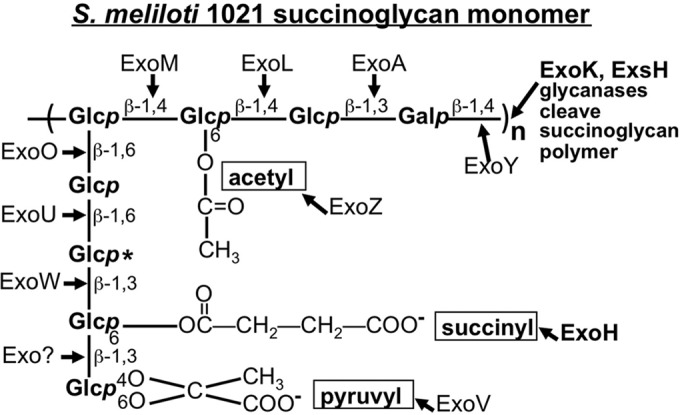
Structure of succinoglycan monomer. Sugar linkages, positions of acetyl, succinyl, and pyruvyl substituents, and the gene product responsible for forming each linkage are shown (33, 34). A second succinylation site is marked with an asterisk (50). Adapted from Mendis et al., 2013 (43).

It is also not known why in *S. meliloti* 1021, succinoglycan is required for infection thread initiation and progression or how it might influence conditions within colonized curled root hairs to facilitate these processes. The succinoglycan monomer is an octasaccharide composed of 1 galactose residue and 7 glucose residues, with acetyl, succinyl, and pyruvyl modifications (33). Negatively charged carboxylates on the succinyl and pyruvyl groups render the polysaccharide acidic. This structure, along with the gene product that catalyzes each step in the biosynthetic pathway, is shown in Fig. 1 (33–37). It has recently been determined that in *Mesorhizobium loti* R7A, an acidic octasaccharide EPS with some structural similarities to succinoglycan interacts with the Epr3 receptor-like kinase in its plant host *Lotus japonicus* (16). An *M. loti* mutant that cannot make this EPS can successfully invade and nodulate *L. japonicus* (13), but *exoU* mutants that produce a truncated pentasaccharide EPS cannot invade wild-type plants (13). Thus, there is a striking difference between the *M. loti-L. japonicus* system and the *S. meliloti-M. truncatula* system: EPS-deficient mutants of *M. loti* can invade their host (13), whereas *S. meliloti* succinoglycan-deficient mutants cannot invade *M. truncatula*. *S. meliloti exoY* and *exoA* mutants, which produce no succinoglycan (9, 34, 38), do not invade host plants (10, 38) and in the case of *exoY* have been shown to fail in initiating infection threads (10).

*S. meliloti* strains that are completely succinoglycan deficient are not the only type of succinoglycan mutant with a symbiotic defect. An *exoH* mutant that produces succinoglycan lacking the succinyl groups (Fig. 1) also cannot invade alfalfa roots (39) and has previously been shown to initiate a reduced number of infection threads on alfalfa and to abort all of the infection threads that are initiated (10). *exoH* mutants produce only the high-molecular-weight (HMW) form because the glycanases ExoK and ExsH cannot cleave the unsuccinylated form (40). A long-standing question about the nature of the defect in *S. meliloti exoH* mutants is whether they fail to invade the host because the succinoglycan they produce is unsuccinylated or because they produce only the HMW form of succinoglycan (40). It has not previously been determined if there is a mechanism independent of ExoK and ExsH cleavage for production of low-molecular-weight (LMW) succinoglycan; however, the existence of alternate routes to the LMW form has been proposed: either through cleavage by another enzyme or through direct export of LMW forms (41, 42). If LMW succinoglycan cannot be produced in the absence of the ExoK and ExsH glycanases, and if LMW succinoglycan is required for infection thread formation, then a double mutant with mutations in both glycanase enzymes should have a very severe symbiotic defect similar to that of strains with a mutation in the *exoH*-encoded succinyltransferase. Conversely, if LMW succinoglycan is not required for infection thread formation, strains deficient in both glycanases should not have a severe symbiotic defect. Thus, it is critical to determine both the symbiotic phenotype of double glycanase mutants and whether or not these strains produce any residual LMW succinoglycan.

We have now characterized the polysaccharides produced by strains deficient in both the ExoK and ExsH glycanases and determined that these strains do not produce any LMW succinoglycan. We have also determined that these “double glycanase” mutants invade *M. truncatula* roots and establish a productive symbiosis, albeit with less efficiency than wild-type *S. meliloti* 1021. This demonstrates that the LMW form of succinoglycan is not required for host invasion. This also indicates that successful symbiosis requires succinylation of succinoglycan for a reason that is independent of the effect of succinylation on susceptibility to glycanase cleavage.

## RESULTS

### Strains deficient in the *exsH*-encoded succinoglycan glycanase do not have a significant reduction in symbiotic productivity.

**FIG 2:**
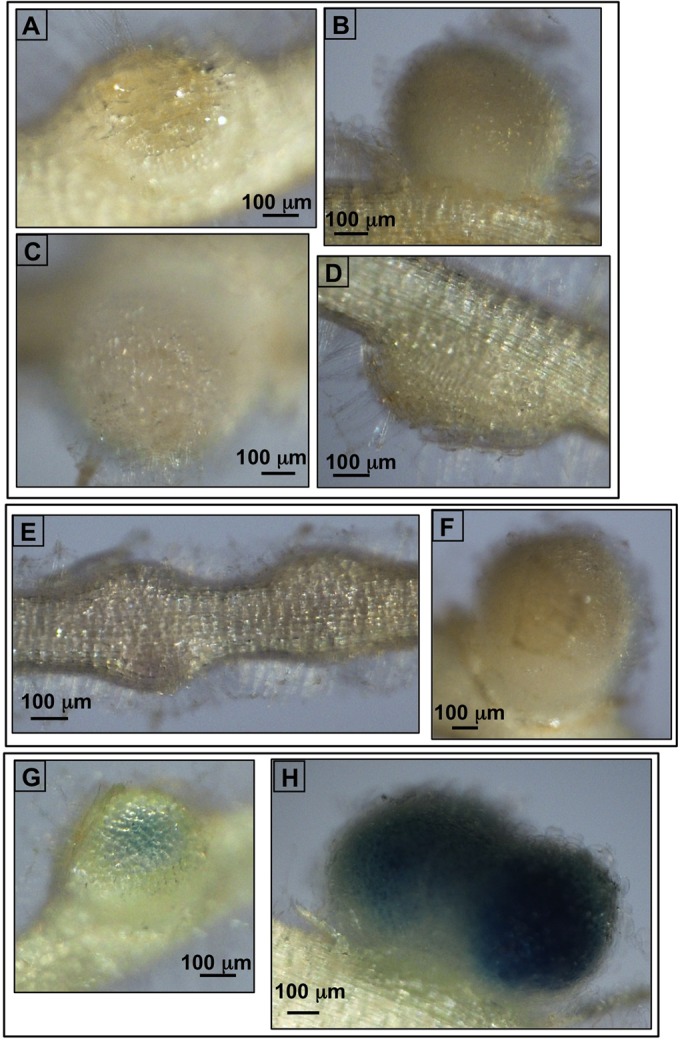
The *exsH*-encoded glycanase is not expressed during invasion and nodulation. (A to D) There is no expression of an *exsH*::β-glucuronidase (GUS) reporter in *S. meliloti* in *M. truncatula* developing nodules at 14 days postinoculation. The GUS reporter is under the transcriptional control of *exsH* upstream elements in strains that also have a complete copy of *exsH* in the genome. Three independently isolated GUS fusion strains are shown: (A) the *exsH*::JH104.7A strain, (B and D) the *exsH*::JH104.12Cstrain, and (C) the *exsH*::JH104.4B strain. All three *exsH*::GUS fusions are expressed when the strains are grown on M9 medium (see Fig. S2 in the supplemental material), demonstrating that the fusions are functional. (E and F) The negative control was *S. meliloti* 1021 without a GUS fusion. (G and H) Positive control for strong GUS expression from an SMc00911::JH104 reporter (70). In all panels, the bar corresponds to 100 µm. Roots were stained with X-Gluc for 48 h.

We had previously constructed a nonpolar deletion mutant of the succinoglycan glycanase-encoding gene *exoK* (see below) and found that this strain can invade *M. truncatula* and form functional nodules, but it does so less efficiently than the wild type (43). To determine if loss of an additional succinoglycan glycanase encoded by *exsH* also has an effect on symbiosis with *M. truncatula*, we tested mutants carrying a Tn*5-233* transposon insertion in *exsH* and found that they do not have a statistically significant defect in symbiotic productivity measured by shoot fresh weight, but they do have a small statistically significant reduction in the number of pink, functional nodules (see Fig. S1 in the supplemental material). The pink color of root nodules induced by rhizobial infection is due to the production of leghemoglobin by the host plant and is indicative of a functional symbiosis (44, 45). This very small effect on the symbiosis is consistent with the lack of detectable expression of the *exsH* glycanase gene in *S. meliloti* during host invasion (Fig. 2). Using strains that carry both an *exsH*::*uidA* β-glucuronidase (GUS) reporter fusion and a complete copy of *exsH* in the genome, we found that expression of *exsH* cannot be detected in *S. meliloti* in infection threads or root nodules at 14 days postinoculation (Fig. 2A to D), a time point at which strong expression of the operon containing *exoK* can be detected (43). GUS is expressed in *exsH* reporter strains when they are grown on M9 medium, demonstrating that the reporter is functional (see Fig. S2 in the supplemental material). Taken together, our results show that expression of the *exsH-*encoded glycanase is not detectable during host invasion, and loss of this glycanase does not have a significant effect on the symbiotic productivity of the association with *M. truncatula*.

### Strains deficient in both the *exoK*- and *exsH-*encoded succinoglycan glycanases invade host roots and form functional nodules.

**FIG 3:**
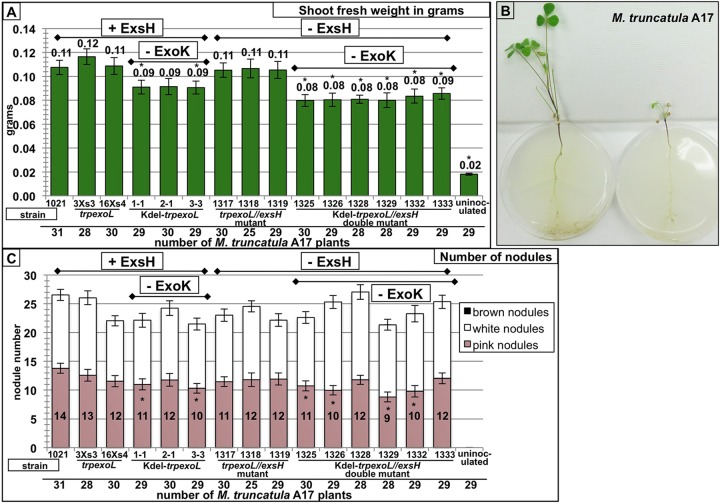
Mutants lacking both ExoK and ExsH glycanases have only a slight reduction in symbiotic productivity and nodulation. (A) Average shoot fresh weight of *M. truncatula* A17 plants inoculated with the *S. meliloti* strain shown. (Multiple independent isolates of each strain were compared. Error bars show standard errors of the mean [SEM] for plants inoculated with each strain.) The ExoK ExsH double glycanase mutants have a small but statistically significant reduction in plant productivity relative to the *S. meliloti* 1021 wild-type strain, to “modified wild-type” *trpexoL* control strains, and to *exsH* single mutants. The symbiotic performance of double glycanase mutants is similar to the symbiotic productivity of Kdel-*trpexoL exoK* single mutants. (B) Representative image of an *M. truncatula* plant inoculated with wild-type *S. meliloti* versus an uninoculated plant. (C) Most Kdel-*trpexoL exoK* single mutants and ExoK ExsH double glycanase mutants also have a small, but statistically significant reduction in the number of mature, pink nodules. (Error bars show SEM.)

ExsH and ExoK are the only glycanases that have been demonstrated to cleave succinoglycan to generate the LMW form in the 1021 strain of *S. meliloti* (41, 46 [also see reference 47]). To determine whether or not LMW succinoglycan is required for successful host invasion of *M. truncatula*, it is necessary to determine the symbiotic phenotype of *exoK exsH* glycanase double mutants and to determine whether or not these double glycanase mutants produce any LMW succinoglycan. The nonpolar *exoK* deletion strains described by Mendis et al. (43) (Kdel-*trpexoL* strains) were constructed as part of a series of strains in which the downstream *exoLAMON* genes are under identical regulatory control despite alterations to the upstream *exoHK* region. The design of these nonpolar *exoK* deletion strains and the “modified wild-type” control strains (*trpexoL* strains) is shown in Fig. S3 in the supplemental material. To construct the double glycanase mutants, we transduced the *exsH*::Tn*5-233* insertion into the Kdel-*trpexoL* strains, generating 6 independently isolated double glycanase mutants (strains 1325, 1326, 1328, 1329, 1332, and 1333). We also transduced *exsH*::Tn*5-233* into the *trpexoL* strains to make *exsH* single mutants in the “modified wild-type” background. Symbiotic phenotypes of double glycanase mutants are shown in Fig. 3, along with symbiotic phenotypes of *exoK* single mutants and *exsH* single mutants. Figure 3A shows average shoot fresh weights of *M. truncatula* plants inoculated with each *S. meliloti* strain. The Kdel-*trpexoL exsH* double glycanase mutants (here, referred to as ExoK ExsH double glycanase mutants) have a small, but statistically significant reduction in plant productivity relative to *S. meliloti* 1021 wild-type and “modified wild-type” *trpexoL* control strains and relative to the *trpexoL exsH* single mutants (Fig. 3A). (Fig. 3B shows a representative *M. truncatula* plant inoculated with wild-type *S. meliloti* 1021 versus an uninoculated plant.) The symbiotic performance of the double glycanase mutants is similar to that of Kdel-*trpexoL* single glycanase mutants (Fig. 3A). Most of the Kdel-*trpexoL* single mutants and the ExoK ExsH double glycanase mutant strains also have a small, but statistically significant reduction in the number of pink, functional nodules (Fig. 3C). These results demonstrate that ExoK ExsH double glycanase mutants, like Kdel-*trpexoL exoK* single mutants, have reduced symbiotic productivity relative to wild-type strains but are still able to form a functional symbiosis on *M. truncatula*.

### Strains deficient in both the ExoK and ExsH succinoglycan glycanases do not produce any detectable LMW succinoglycan.

**FIG 4:**
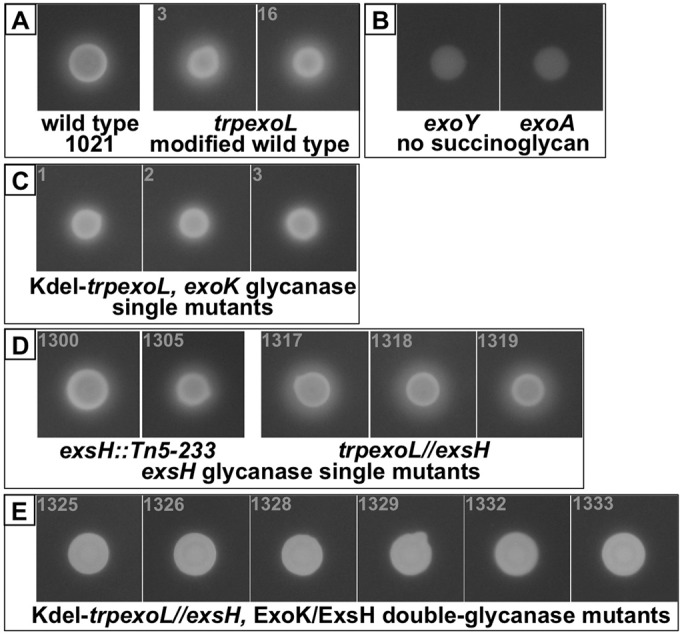
There is no LMW succinoglycan detectable from ExoK ExsH double glycanase mutants with the Calcofluor-fluorescence halo assay. After 12 days of growth on GMS medium containing 0.02% Calcofluor, *S. meliloti* 1021 wild-type and *trpexoL* “modified wild-type” strains (A) produce a large halo of diffused LMW succinoglycan. Negative-control, succinoglycan-deficient *exoY* and *exoA* strains (B) are “Calcofluor dark,” showing that they do not produce succinoglycan. (C) Kdel-*trpexoL exoK* single mutants have a reduced halo diameter, consistent with production of an intermediate amount of LMW succinoglycan. (D) *exsH* single mutants in both the 1021 and the *trpexoL* backgrounds have halos of similar size to the wild type, suggesting that loss of *exsH* has little effect on production of LMW succinoglycan. (E) ExoK ExsH double glycanase mutants produce no detectable Calcofluor halo, suggesting that no LMW succinoglycan is produced in the absence of these two glycanases.

In order to determine if the LMW form of succinoglycan is required for successful symbiosis by *S. meliloti*, it is necessary to determine whether or not these symbiosis-functional ExoK ExsH double glycanase mutants produce any LMW succinoglycan. We had previously determined that Kdel-*trpexoL exoK* single mutants produce a reduced but substantial amount of LMW succinoglycan that can be seen in an LMW succinoglycan-diffusion “halo” assay using the fluorescent dye Calcofluor (43). To determine if the ExsH glycanase is the source of the LMW succinoglycan in these Kdel-*trpexoL* single mutants, we compared the ExoK ExsH double glycanase mutants with the single glycanase mutants and control strains in a Calcofluor halo assay (Fig. 4). We found that after 12 days of growth on GMS (glutamate mannitol salts medium) medium containing 0.02% Calcofluor, ExoK ExsH double glycanase mutants did not produce a visible halo of LMW succinoglycan (Fig. 4E). This contrasts with the abundant LMW succinoglycan produced by *S. meliloti* 1021 wild-type and *trpexoL* “modified wild-type” strains (Fig. 4A). It also contrasts with the large amount of LMW material made by *exsH* single mutants in both the 1021 and the *trpexoL* backgrounds (Fig. 4D) and the intermediate amount of LMW material already demonstrated to be made by Kdel-*trpexoL exoK* single mutants (43) (Fig. 4C). Thus, it appears that the majority of LMW succinoglycan is made by ExoK cleavage of the polymer with some contribution by ExsH cleavage that is apparent in the absence of ExoK.

It had previously been determined in another study (41) that strains carrying transposon insertions in both *exoK* and *exsH* do not produce an LMW succinoglycan “halo” in a Calcofluor assay, but that these strains do produce a residual amount of LMW polysaccharide material that can be detected with the anthrone-sulfuric acid assay for hexose sugars (48). However, the identity of the LMW, hexose-containing material produced by these strains was not established in this previous study. In order to determine whether any of the LMW polysaccharide produced by ExoK ExsH double glycanase mutants is succinoglycan, we characterized LMW polysaccharide from these strains by size separation and sugar composition analysis. We isolated culture supernatant from GMS minimal medium cultures of wild-type 1021 and two independently isolated ExoK ExsH double glycanase mutants, 1325 and 1328. Culture supernatant from the succinoglycan-deficient *exoY* mutant (34) served as a negative control. We used total culture supernatant rather than alcohol-precipitated polysaccharide because it has been reported that alcohol precipitation is inefficient in isolating LMW forms of succinoglycan (49). Table 1 shows the quantification of polysaccharide calculated from the anthrone-positive material per milliliter from each culture supernatant normalized to the cell density of the culture measured at optical density at 600 nm (OD_600_). The two ExoK ExsH double glycanase mutant strains tested, 1325 and 1328, produce 60 to 70% of the amount of polysaccharide produced by the wild type (Table 1). In contrast, the succinoglycan-deficient *exoY* mutant produces 10% of the amount of polysaccharide produced by the wild type. This demonstrates that even the *exoY* mutant produces a small amount of hexose-containing polysaccharide, while the wild-type and the double glycanase mutants produce a large quantity of hexose-containing material.

**TABLE 1:** EPS production by the *S. meliloti* wild type and mutants

Strain	Amt of anthrone-positive material normalized to culture density OD_620_ of anthrone per ml/OD_600_ cell density	% of total anthrone-positive material of <10 kDa
*S. meliloti* 1021 wild type	5.00	70
*exoY*::Tn*5* mutant (no succinoglycan)	0.46 (10% of wild type)	40
1325 ExoK ExsH double glycanase mutant	2.81 (60% of wild type)	40
1328 ExoK ExsH double glycanase mutant	3.57 (70% of wild type)	30

LMW succinoglycan produced by wild-type *S. meliloti* 1021 has previously been determined to consist of monomers, dimers, and trimers of the octasaccharide (42, 50) with calculated molecular masses of 1.5 to 1.7, 3.1 to 3.5, and 4.6 to 5.2 kDa, respectively. (The molecular mass range is due to variability in degree of succinylation of each succinoglycan monomer.) In order to isolate LMW material, we collected solutes smaller than 10 kDa in size by filtering the culture supernatants through a 10-kDa molecular mass cutoff (MMCO) membrane. Table 1 shows the percentage of total polysaccharide produced by each strain that is smaller than 10 kDa in size. The succinoglycan-deficient *exoY* mutant and both ExoK ExsH double glycanase mutants produce similar percentages (30 to 40%) of total polysaccharide as species smaller than 10 kDa, while the wild type produces a much greater percentage of total polysaccharide in forms smaller than 10 kDa (70%).

**FIG 5:**
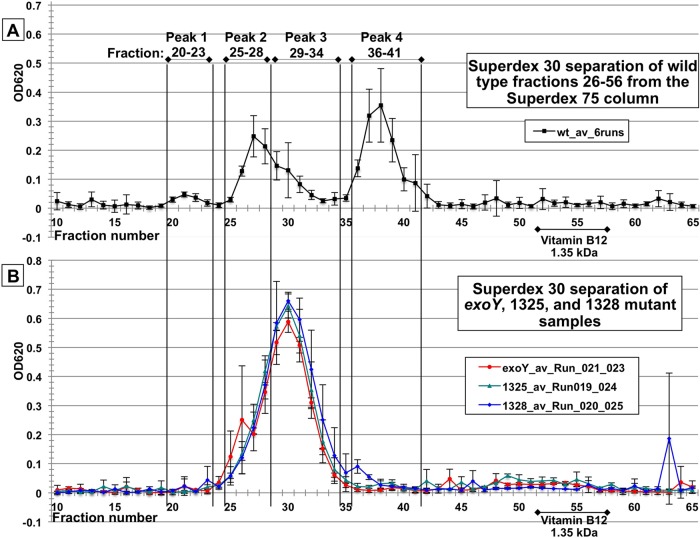
Separation of *S. meliloti* polysaccharides on a Superdex 30 column. (A) For each of the 6 wild-type sample aliquots fractionated on the Superdex 75 column, fractions 26 to 56 were pooled, concentrated, and separated individually on a Superdex 30 column. This material resolved into 4 peaks, discussed in the main text. Error bars show SEM for the average of 6 column runs. Vitamin B_12_ (1.35 kDa) served as an LMW marker in all column runs. (B) Separation of the <10-kDa polysaccharide material from the succinoglycan-deficient *exoY* mutant and ExoK ExsH double glycanase mutants 1325 and 1328. This material resolved into a single major peak centered on fraction 30. Error bars show SEM for the average of 3 column runs for each strain.

The LMW fraction of each sample was further fractioned by size exclusion on a Superdex 75 column and the hexose sugar content of the fractions analyzed by anthrone-sulfuric acid assays. The results are shown in Fig. S4 in the supplemental material. Wild-type LMW polysaccharide from the Superdex 75 column was collected for finer fractionation on a Superdex 30 column. Wild-type LMW material was resolved on the Superdex 30 column (Fig. 5A) into 2 major peaks composed of fractions 25 to 28 (peak 2) and fractions 36 to 41 (peak 4) and 2 minor peaks of fractions 20 to 23 (peak 1) and fractions 29 to 34 (peak 3) (Fig. 5A). Vitamin B_12_ (1.35 kDa) served as LMW marker in all column runs (Fig. 5). Samples of succinoglycan-deficient *exoY* mutant and ExoK ExsH double glycanase mutants 1325 and 1328 were also separated on the Superdex 30 column (Fig. 5B). What appears to be a single large polysaccharide peak was detected at an identical position in fractions 25 to 34 in both ExoK ExsH double glycanase mutants and in the succinoglycan-deficient *exoY* mutant. This peak is centered on fraction 30, which is the same position as peak 3 from the wild type. The fact that ExoK ExsH double glycanase mutants have an LMW polysaccharide profile nearly identical to the succinoglycan-deficient *exoY* mutant suggests that LMW polysaccharides produced by the ExoK ExsH double glycanase mutants are not succinoglycan.

In order to establish the identity of these peaks, the glucose/galactose ratio of each peak was determined. Sugar composition of the peaks from fractions 25 to 28 (peak 2), 29 to 34 (peak 3), and 36 to 41 (peak 4) from wild-type *S. meliloti* 1021 and of the peaks centered on fraction 30 from the mutants was determined by the alditol acetate method at the University of Georgia Complex Carbohydrate Research Center, and the results are summarized in Table 2. The succinoglycan monomer has previously been determined by mass spectrometry to contain 7 glucose sugars and 1 galactose sugar (33). In contrast, cyclic β-glucans are pure glucose, and in *S. meliloti* are close in size to the succinoglycan dimer (51). The other polysaccharide that may be produced by *S. meliloti* 1021 in very small quantities, EPSII (also known as galactoglucan) has a repeating unit of 1 galactose:1 glucose (52, 53). The sugar composition analysis described below indicates that the peaks from the wild type are composed chiefly of succinoglycan, while the peaks centered on fraction 30 from the *exoY* mutant and from the ExoK ExsH double glycanase mutants are cyclic β-glucans.

**TABLE 2:** Glycosyl composition of LMW polysaccharide fractions

Strain	µg glucose equivalents of hexose sugars (anthrone/sulfuric acid assay)^a^			mol% of glycosyl residues in sample^a^^,^^b^		
	Fractions 25–28	Fractions 29–34	Fractions 36–41	Fractions 25–28	Fractions 29–34	Fractions 36–41
*S. meliloti* 1021 wild type	142 (peak 2)	92 (peak 3)	316 (peak 4)	80.0% glucose, 16.6% galactose	88.6% glucose, 8.0% galactose	81.3% glucose, 16.4% galactose
*exoY*::Tn*5* mutant (no succinoglycan)	171	497	12	96.5% glucose, 0.5% galactose	94.7% glucose, 3.4% galactose	ND^c^
1325 ExoK ExsH double glycanase mutant	189	450	13	98.4% glucose, 0.7% galactose	100.0% glucose, 0% galactose	ND
1328 ExoK ExsH double glycanase mutant	288	674	27	97.8% glucose, 0% galactose	94.9% glucose, 1.6% galactose	ND

a The fraction numbers shown are from the Superdex 30 column.

b Some samples contained <2.2% each mannose, xylose, and/or arabinose. Full glycosyl composition results are shown in Table S2 in the supplemental material.

c ND, not determined (no polysaccharide peak).

Wild-type fractions 25 to 28 (peak 2) contain 80.0% glucose and 16.6% galactose with small quantities of other sugars (for full results, see Table S2 in the supplemental material), which is a glucose/galactose ratio of ~5:1. Wild-type fractions 36 to 41 contain 81.3% glucose and 16.4% galactose, which is also a glucose/galactose ratio of 5:1. Although a glucose/galactose ratio of 7:1 rather than 5:1 is predicted based on succinoglycan structure (33), a 5:1 ratio is very close to values previously detected for succinoglycan monomer by the alditol acetate method (e.g., see Fig. 1 in reference 42, in which the monomer peak glucose/galactose ratio was ~5.5:1) (42). In contrast, wild-type fractions 29 to 34 (peak 3) contain 88.6% glucose and 8% galactose, which is a glucose/galactose ratio of 11:1 and is also similar to the value previously determined for peaks that are a mixture of succinoglycan and cyclic β-glucans (42). Based on the elution positions of Superdex 30 column peaks, sugar composition analysis, and comparisons with earlier work (42, 50), we conclude that wild-type fractions 25 to 28 (peak 2) are succinoglycan dimer, fractions 29 to 34 (peak 3) are a mixture of cyclic β-glucans and succinoglycan, and fractions 36 to 41 (peak 4) are succinoglycan monomer. The sugar composition of fractions 20 to 23 (peak 1) was not tested since it has such a small amount of hexose-positive material, but its elution position is consistent with succinoglycan trimer. Compared with previous observations, we isolated a smaller quantity of trimer relative to the quantity of dimer and monomer (50). One possible explanation for this is that by not alcohol precipitating polysaccharide, we retained a larger percentage of the dimer and monomer present in the culture supernatant. This is consistent with the observation that precipitation of LMW succinoglycan is inefficient (49).

LMW polysaccharide material from the succinoglycan-deficient *exoY* mutant and from both ExoK ExsH double glycanase mutants appears to be a single broad peak centered on fraction 30. However, based on the Superdex 30 separation alone, we could not exclude the possibility that there were multiple peaks in these fractions representing multiple hexose-containing species. Therefore, we separately analyzed fractions 25 to 28 and fractions 29 to 34 as we had done for the peaks from the wild type. The results are summarized in Table 2. The material from fractions 25 to 28 and from fractions 29 to 34 from ExoK ExsH double glycanase mutants are ≥95% glucose and ≤1.6% galactose, indicating that these mutants do not produce a significant amount of galactose-containing polysaccharide of this size. In fact, these fractions from the ExoK ExsH double glycanase mutants have less galactose than the same fractions from the succinoglycan-deficient *exoY* mutant, which strongly suggests that these mutants do not produce any LMW succinoglycan. The size of oligosaccharides from all these mutants and the fact that they are composed almost exclusively of glucose, strongly suggest that they are cyclic β-glucans.

### Strains deficient in the *exoH-*encoded succinyltransferase and both succinoglycan glycanases cannot engage in a productive symbiosis.

**FIG 6:**
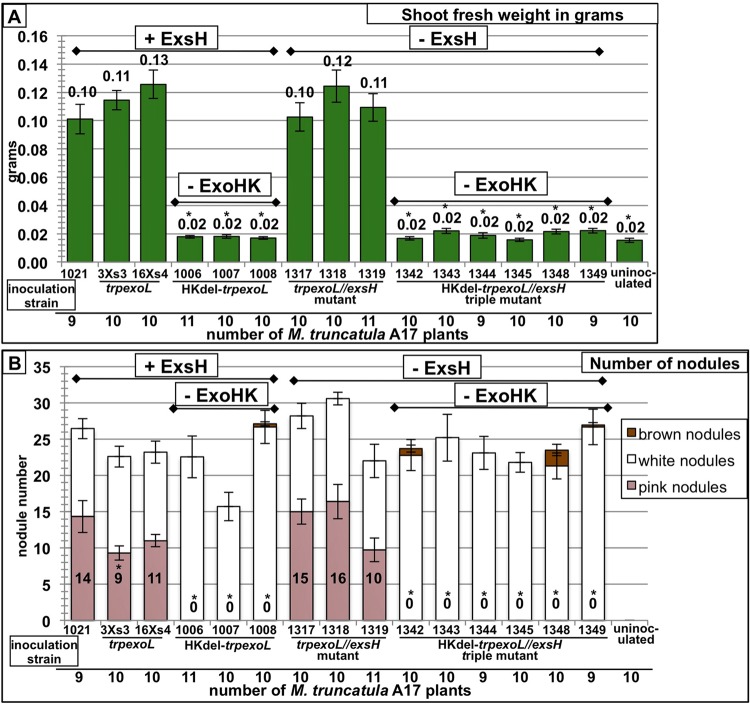
Triple mutants lacking ExoK and ExsH glycanases and the *exoH*-encoded succinyltransferase cannot form a functional symbiosis. (A) Average shoot fresh weight of *M. truncatula* A17 plants inoculated with the *S. meliloti* strain shown on the label. Growth of plants inoculated with any of 6 independently isolated triple mutant strains (1342, 1343, 1344, 1345, 1348, and 1349) on nitrogen-free medium is completely arrested and is indistinguishable from that of uninoculated plants. (B) Plants inoculated with triple mutants form only small, white, nonfunctional nodules. (Error bars show SEM.) The number of plants inoculated with each strain is shown.

In order to determine whether succinylation of succinoglycan is the critical factor in its symbiotic function, we made strains that are isogenic to the ExoK ExsH double glycanase mutants, except that they are also deleted for the *exoH*-encoded succinyltransferase. (For deletion design, see Fig. S3 in the supplemental material.) The symbiotic phenotypes of these triple mutant strains that lack both glycanases and the succinyltransferase are shown in Fig. 6. Growth of plants inoculated with any of 6 independently isolated, triple mutants (strains 1342, 1343, 1344, 1345, 1348, and 1349) on nitrogen-free medium is completely arrested and is indistinguishable from that of uninoculated plants (Fig. 6A). These plants form only small, nonfunctional nodules (Fig. 6B). When *exoH* is introduced on a plasmid into the triple mutants, symbiotic performance is restored to the level of the ExoK ExsH double glycanase mutants (data not shown). Introduction of a plasmid carrying *exoK* into the triple mutants has no effect on symbiosis (data not shown).

Compared with the symbiotic performance of the ExoK ExsH double glycanase mutants that produce no LMW succinoglycan, but still have the *exoH* succinyltransferase, the difference is striking. Loss of the *exoH-*encoded succinyltransferase results in a complete inability to form functional nodules and to support host plant growth, while strains that lack only the glycanases have merely a slight reduction in symbiotic productivity. This demonstrates that succinylation of succinoglycan is required for *S. meliloti* to engage in a functional symbiosis and that this requirement is independent of the effect that succinylation has on susceptibility to glycanase cleavage.

## DISCUSSION

We have demonstrated that *S. meliloti* 1021 double mutants deficient in both ExoK and ExsH glycanases make only HMW succinoglycan and that these strains can form a productive symbiosis with the host *M. truncatula*. This is the first report providing biochemical evidence that the residual LMW hexose-sugar-containing material produced by *S. meliloti* 1021 ExoK ExsH double glycanase mutants is not succinoglycan but is most likely cyclic β-glucans. This demonstrates that, at least under these growth conditions, production of LMW succinoglycan is completely dependent upon ExoK and ExsH glycanases. Our results also demonstrate that ExoK ExsH double glycanase mutants can form a productive symbiosis with *M. truncatula*, which in this case must be mediated by HMW succinoglycan. In studies that report partial rescue of host invasion by an *S. meliloti* succinoglycan-deficient mutant by coinoculation with succinoglycan, it was the LMW succinoglycan fraction that promoted rescue (50, 54, 55). These findings are not mutually exclusive with our results. A requirement for the LMW form in rescue studies could be specific to experiments in which succinoglycan is exogenously applied to the root hair surface and is not actively being secreted by bacteria in infection threads.

Other symbiotic EPSs can mediate infection thread formation by other strains of *Sinorhizobium meliloti* (19), but these other polysaccharides are not produced in *S. meliloti* 1021. For example, an *expR101* mutant of *S. meliloti* 1021, *S. meliloti* 8530, produces the EPS galactoglucan (EPSII) in sufficient quantities to mediate infection thread formation on the host alfalfa (19). However, in *S. meliloti* 1021, under phosphate-replete conditions, EPSII is produced in, at most, trace amounts (56–58). Also, EPSII cannot support invasion on the host *M. truncatula* (21). Therefore, EPSII cannot have been responsible for host invasion of *M. truncatula* by the ExoK ExsH double glycanase mutants. In addition, the capsular K antigen of *S. meliloti* strain Rm41 can also mediate infection thread formation on the host alfalfa (19), but the *S. meliloti* 1021 strain lacks fully functional paralogs of the critical Rm41 *rkpZ* gene and consequently cannot produce K antigen in a symbiotically functional form (23, 59, 60). Therefore, infection thread formation on *M. truncatula* by *S. meliloti* 1021 is dependent on succinoglycan, and successful host invasion by the ExoK ExsH double glycanase mutants is not mediated by EPSII or K antigen.

This work also answers the long-standing question regarding whether *S. meliloti* strains with a mutation in the *exoH*-encoded succinyltransferase fail to form a productive symbiosis because the succinoglycan they produce is unsuccinylated or, instead, because it cannot be cleaved by the glycanases and is therefore only in the HMW form. ExoK ExsH double glycanase mutants producing HMW succinylated succinoglycan form a successful symbiosis with *M. truncatula*, while triple mutants with mutations in both glycanases and *exoH*, producing HMW unsuccinylated succinoglycan, form neither functional nodules nor a productive symbiosis. Since we have shown that the successful ExoK ExsH double glycanase mutants do not produce LMW succinoglycan, this strongly suggests that the symbiotic defect in *exoH* succinyltransferase-deficient mutants is due to the lack of the acidic succinyl group on succinoglycan.

The unsuccinylated succinoglycan produced by an *exoH* mutant of *S. meliloti* lacks 1 to 2 negatively charged substituents per monomer, although it retains the negatively charged pyruvyl group (39). The degree of succinylation of EPS produced by other *S. meliloti* strains has also been proposed to affect the ability of each strain to form a productive symbiosis on a particular *M. truncatula* ecotype (61). The loss of the succinyl groups of succinoglycan results in an increase in viscosity and in polymer chain stiffness, probably due to reduced charge density (62). It is also likely that loss of negative charge would reduce the ability of succinoglycan to interact with positively charged ions in the infection thread matrix (28). Another possibility is that modifications to succinoglycan could alter its ability to quench reactive oxygen species (ROS) in the infection thread (63). These factors might affect the fluidity of the infection thread matrix, thereby affecting infection thread progression (5, 64).

Recent studies on the EPS produced by the *exoU* mutant of *Mesorhizobium loti* R7A also suggest that loss of negative charge on symbiotically active rhizobial EPSs may be important for function (13, 16). The truncated, pentasaccharide EPS produced by the *exoU* mutant lacks one neutral glucose sugar and two negatively charged uronic acid sugars (glucuronic acid and riburonic acid) (13, 16). This truncated *M. loti exoU* mutant-produced EPS prevents infection thread formation on the host plant *L. japonicus*, and this blockage is dependent upon the *L. japonicus* Epr3-encoded receptor-like kinase (13, 16). It is not yet known if it is the loss of negative charge from uronic acids or other structural features of the truncated *exoU* mutant EPS that is critical for blocking infection thread formation. It is possible that *S. meliloti* succinoglycan similarly interacts with an *M. truncatula* ortholog of EPR3 and that loss of the negatively charged succinyl groups leads to a blockage in infection thread formation.

An important difference between the roles of EPS in *M. loti-L. japonicus* symbiosis and *S. meliloti-M. truncatula* symbiosis is that an *M. loti* EPS-deficient *exoB* mutant can invade *L. japonicus* roots and form a functional symbiosis, although it is less efficient, inducing 30 to 50% the number of mature, extended infection threads induced by the wild type at 10 to 14 days postinoculation (13, 16). This contrasts with the requirement for succinoglycan in infection thread formation on plant hosts by *S. meliloti*. A succinoglycan-deficient *S. meliloti exoY* mutant forms no extended infection threads on the host alfalfa by 10 to 12 days postinoculation (10). This is consistent with earlier work showing that EPS production by rhizobia is more critical for symbiosis on plant hosts that form indeterminate nodules, such as alfalfa and *M. truncatula*, than on those that form determinate nodules, such as *L. japonicus* (65). In indeterminate nodules, infection threads must be maintained throughout the life of the nodule to allow bacteria to reach and invade not only cells of the nodule primordium but also the newly divided plant cells behind the persistent nodule meristem (7). The accumulation of aborted infections (10, 66), cytological evidence for plant defense responses (67), and expression of plant defense genes (66) in roots inoculated with succinoglycan-deficient strains of *S. meliloti* provide extensive evidence for a role for succinoglycan in the intimate interaction between bacteria and root cells during invasion. Whether all of these critical symbiotic interactions between succinoglycan and its host are dependent upon an *M. truncatula* ortholog of *L. japonicus* EPR3 receptor-like kinase remains to be determined.

## MATERIALS AND METHODS

### Bacterial strains and growth conditions.

*S. meliloti* 1021 strains (see Table S1 in the supplemental material) were grown at 30°C in LBMC medium (68), GMS (glutamate mannitol salts medium) (41), M9 minimal medium (8), or Jensen’s plant medium with glutamate and mannitol (27). Bacterial plates contained 1.5% Bacto agar (BD, Franklin Lakes, NJ). Calcofluor polysaccharide indicator plates contained 0.02% Calcofluor white M2R (fluorescent brightener 28 [Sigma, St. Louis, MO]) (8). The antibiotic concentrations were 1 mg/ml or 500 µg/ml streptomycin, 200 µg/ml neomycin, 25 µg/ml gentamicin, and 50 µg/ml spectinomycin.

### Construction of plasmids and *S. meliloti* mutant strains.

Restriction enzymes and polymerases were obtained from New England Biolabs (Ipswich, MA). Primers were obtained from Eurofins MWG Operon (Huntsville, AL). Transductions were performed using phage φM12 (69). All strains, plasmids, and primers and the construction of strains are described in Table S1 in the supplemental material.

### Plant nodulation assays.

Host plant *Medicago truncatula* cv. “Jemalong A17” was prepared for inoculation with *S. meliloti* as previously described (68). Seedlings were moved to individual Jensen’s medium microcosms and inoculated with *S. meliloti* of the appropriate strain as described previously (68). Plants were grown in a Percival AR-36L incubator (Perry, IA) at 21°C, with 60 to 70% relative humidity and 100 to 175 µmol m^−2^ s^−1^ light for 7 weeks.

### Detection of β-glucuronidase activity and imaging of roots and nodules.

β-Glucuronidase expression by bacteria was detected by staining whole roots in X-Gluc buffer (1 mM 5-bromo-4-chloro-3-indolyl-β-d-glucuronic acid, cyclohexylammonium salt; 0.02% SDS, 50 mM Na-phosphate [pH 7]) (70) for 48 h. Whole roots were imaged on an AZ100 Multi-Zoom microscope equipped with a DS-Fi1, 5-megapixel color camera (Nikon Instruments, Melville, NY).

### Polysaccharide fractionation and quantification.

To isolate LMW polysaccharides, total culture supernatant from 5-day GMS cultures was collected by centrifugation for 20 min at 11,000 × *g* in a Beckman Avanti J-20XP centrifuge. Hexose-sugar-containing polysaccharide was quantified by anthrone-sulfuric acid assays as described previously (27). The optical density at 620 nm (OD_620_) of sample anthrone assays was compared to a 2-fold dilution series of glucose. Anthrone assays performed on the appropriate medium served as the blank. Culture supernatant was then vacuum filtered through a 0.2-µm-pore filter, followed by pressure filtration through a 10-kDa filter in a stirred cell. After isolation of material of ≤10 kDa, samples were freeze-dried and resuspended in deionized water. Insoluble material was removed by centrifugation. Samples were dialyzed first against deionized water and then against 0.125 M NaCl–0.125 M Na-acetate, using a 0.5- to 0.1-kDa MMCO membrane (Spectrum Labs, Rancho Dominguez, CA).

Following dialysis, samples were fractionated further by size exclusion chromatography performed on an AKTA Purifier (GE Healthcare). Wild-type sample aliquots were normalized to 50 µg total protein and loaded in 0.85 ml onto a HiLoad 16/60 Superdex 75 size exclusion column (GE Healthcare). The Superdex 75 column was run in 0.125 M NaCl–0.125 M Na acetate buffer at 0.5 ml/min. Fractions of 1.4 ml were collected with 0.25 column volume discarded prior to the start of fractionation. Vitamin B_12_ (1.35 kDa) was included as a low-molecular-mass marker. The hexose-sugar content of each fraction was measured by anthrone-sulfuric acid assays.

To further resolve LMW peaks from the wild type, fractions 26 to 56 of each of 6 Superdex 75 column runs of wild-type sample were concentrated by freeze-drying and dialyzed against 0.125 M NaCl–0.125 M Na acetate using a 0.5- to 0.1-kDa MMCO membrane. The pooled fractions from each Superdex 75 run were individually loaded in 1 ml onto a HiLoad 16/60 Superdex 30 size exclusion column (GE Healthcare). The Superdex 30 column was run in 0.125 M NaCl–0.125 M Na acetate buffer at 0.5 ml/min. Fractions of 1.2 ml were collected with 0.25 column volume discarded prior to starting fractionation. The average total OD_620_ value measured by the anthrone-sulfuric acid assay for Superdex 30 fractions 10 to 65 from the wild type was 3.26. Sample from the *exoY* mutant and double glycanase mutants 1325 and 1328 that had been size selected between 10 and 0.5 kDa (as described above) was diluted to 3.26 OD_620_ anthrone assay units per ml. One-milliliter aliquots were run on the Superdex 30 column under the same conditions as the wild type.

### Glycosyl composition analysis.

Fractions 25 to 28 from all wild-type samples run on the Superdex 30 column were pooled and dialyzed against deionized water using a 1- to 0.5-kDa MMCO membrane and freeze-dried. Fractions 29 to 34 and fractions 36 to 41 from all wild-type samples were similarly pooled and dialyzed using a 0.5- to 0.1-kDa MMCO membrane. The same fraction pools were prepared from the *exoY* mutant and from double glycanase mutants 1325 and 1328. Glycosyl composition analysis was performed by combined gas chromatography-mass spectrometry (GC/MS) of alditol acetates (AAs) as previously described (71) at the University of Georgia Complex Carbohydrate Research Center. Composition analysis was performed using 300 to 500 µg of sample. As the internal standard, 20 µg inositol was added to samples. Samples were hydrolyzed in 2 M trifluoroacetic acid (TFA) for 2 h in a sealed tube at 121°C, reduced with NaBD_4_, and acetylated using acetic anhydride-TFA. The resulting AAs were analyzed on an Agilent 7890A gas chromatograph interfaced with a 5975C MSD in electron impact ionization mode. Separation was performed on a 30-m Supelco SP-2331 bonded-phase fused silica capillary column.

## SUPPLEMENTAL MATERIAL

Text S1  Supplemental references. Download Text S1, DOCX file, 0.1 MB

Figure S1  *exsH* single mutants do not have a reduction in symbiotic productivity, but they have a slight reduction in the number of mature, functional nodules. (A) Strains carrying a Tn*5-233* or Tn*5* insertion in the *exsH* glycanase do not have a statistically significant reduction of symbiotic productivity. (B) The same *exsH*::Tn*5-233* strains shown in panel A have a small, statistically significant reduction in the number of pink, functional nodules relative to the *S. meliloti* 1021 wild type. For both panels A and B, the error bars show SEM for plants inoculated with each strain. The number of plants inoculated with each strain is shown. Download Figure S1, TIF file, 2.4 MB

Figure S2  Expression of an *exsH*::β-glucuronidase (GUS) reporter in *S. meliloti* on M9 minimal medium plates. The GUS reporter in the inserted plasmid pJH104 is under the transcriptional control of the *exsH* upstream elements. (A and B) Three independently isolated GUS fusion strains are shown after 13 days of growth: the *exsH*::JH104.7A, *exsH*::JH104.12C, and *exsH*::JH104.4B strains. Expression is compared to that of the negative control, *S. meliloti* 1021 without a GUS fusion, and the positive control for strong GUS expression from a *greA*::JH104 reporter fusion. (C and D) There is no expression of an *exsH*::β-glucuronidase reporter fusion in the same *S. meliloti* strains on GMS plates after 13 days of growth (Some *exsH*::β-glucuronidase expression was apparent on GMS plates after 5 weeks [data not shown].) (E and F) There is no expression of an *exsH*::β-glucuronidase reporter in the same *S. meliloti* strains on plates containing Jensen’s medium plus glutamate and mannitol plant medium after 13 days of growth. (No expression is apparent after 5 weeks of growth [data not shown].) Download Figure S2, TIF file, 2.4 MB

Figure S3  Design of the *trpexoL* series of strains with modified regulation of the *exoHKLAMON* operon. The *trpexoL*, Kdel-*trpexoL*, and HKdel-*trpexoL* strains have a neomycin-resistance cassette and a *Salmonella* trp promoter, which is constitutively expressed in *S. meliloti*, separating the *exoHK* genes from the *exoLAMON* genes. This provides identical regulatory control of the *exoLAMON* genes in each of the strains. *trpexoL* strains are “modified wild type” and serve as a controls for the modifications. Kdel-*trpexoL* strains have *exoK* deleted, and HKdel-*trpexoL* strains have *exoH* and *exoK* deleted. Download Figure S3, TIF file, 2.4 MB

Figure S4  Separation of *S. meliloti* 1021 polysaccharides on a Superdex 75 column. (A and B) Separation of the <10-kDa polysaccharide material from (A) the wild type and (B) the *exoY* mutant and the ExoK ExsH ExoK ExsH double glycanase mutants. The wild-type sample contains some HMW material that is excluded in the void volume, while material of this size is not apparent in the *exoY* or ExoK ExsH double glycanase mutant samples. Fractions 26 to 56 from the Superdex 75 column were collected for finer fractionation on a Superdex 30 column. Error bars in panel A show the SEM for each data point in 5 replicate column runs. Download Figure S4, TIF file, 2.4 MB

Table S1  Strains, plasmids, and primers used in this study. Table S1, DOCX file, 0.2 MB

Table S2  Full results of the glycosyl composition analysis. Table S2, DOCX file, 0.1 MB
