# Supplementary material for: Function of Succinoglycan Polysaccharide in Sinorhizobium meliloti Host Plant Invasion Depends on Succinylation, Not Molecular Weight
Source: mBio. 2016 Jun 21;7(3):e00606-16. doi: 10.1128/mBio.00606-16 (PMC4916376; doi:10.1128/mBio.00606-16)
Supplement: Text S1 — Supplemental references. Download [file mbo003162857s1.docx]

**Supplemental Material References.**

1. **Backman K, Boyer HW.** 1983. Tetracycline resistance determined by pBR322 is mediated by one polypeptide. Gene **26:**197-203.

2. **Finan TM, Kunkel B, de Vos GF, Signer ER.** 1986. Second symbiotic megaplasmid in *Rhizobium meliloti* carrying exopolysaccharide and thiamine synthesis genes. J Bacteriol **167:**66-72.

3. **Meade HM, Long SR, Ruvkun GB, Brown SE, Ausubel FM.** 1982. Physical and genetic characterization of symbiotic and auxotrophic mutants of *Rhizobium meliloti* induced by transposon Tn5 mutagenesis. J Bacteriol **149:**114-122.

4. **Cheng HP, Walker GC.** 1998. Succinoglycan is required for initiation and elongation of infection threads during nodulation of alfalfa by *Rhizobium meliloti*. J Bacteriol **180:**5183-5191.

5. **Mendis HC, Queiroux C, Brewer TE, Davis OM, Washburn BK, Jones KM.** 2013. The succinoglycan endoglycanase encoded by exoK is required for efficient symbiosis of Sinorhizobium meliloti 1021 with the host plants Medicago truncatula and Medicago sativa (Alfalfa). Mol Plant Microbe Interact **26:**1089-1105.

6. **York GM, Walker GC.** 1997. The *Rhizobium meliloti exoK* gene and *prsD*/*prsE*/*exsH* genes are components of independent degradative pathways which contribute to production of low-molecular-weight succinoglycan. Mol Microbiol **25:**117-134.

7. **Queiroux C, Washburn BK, Davis OM, Stewart J, Brewer TE, Lyons MR, Jones KM.** 2012. A comparative genomics screen identifies a Sinorhizobium meliloti 1021 sodM-like gene strongly expressed within host plant nodules. BMC Microbiol **12:**74.

8. **Ferguson GP, Datta A, Carlson RW, Walker GC.** 2005. Importance of unusually modified lipid A in *Sinorhizobium* stress resistance and legume symbiosis. Mol Microbiol **56:**68-80.

9. **Quandt J, Hynes MF.** 1993. Versatile suicide vectors which allow direct selection for gene replacement in gram-negative bacteria. Gene **127:**15-21.

10. **Fellay R, Frey J, Krisch H.** 1987. Interposon mutagenesis of soil and water bacteria: a family of DNA fragments designed for in vitro insertional mutagenesis of Gram-negative bacteria. Gene **52:**147-154.

11. **Glazebrook J, Walker GC.** 1991. Genetic techniques in Rhizobium meliloti. Methods Enzymol **204:**398-418.

12. **Jones KM.** 2012. Increased production of the exopolysaccharide succinoglycan enhances Sinorhizobium meliloti 1021 symbiosis with the host plant Medicago truncatula. J Bacteriol **194:**4322-4331.

13. **Egelhoff TT, Long SR.** 1985. *Rhizobium meliloti* nodulation genes: identification of *nodDABC* gene products, purification of nodA protein, and expression of *nodA* in *Rhizobium meliloti*. J Bacteriol **164:**591-599.
