# Supplementary figures and images for: Function of Succinoglycan Polysaccharide in Sinorhizobium meliloti Host Plant Invasion Depends on Succinylation, Not Molecular Weight
Source: mBio. 2016 Jun 21;7(3):e00606-16. doi: 10.1128/mBio.00606-16 (PMC4916376; doi:10.1128/mBio.00606-16)

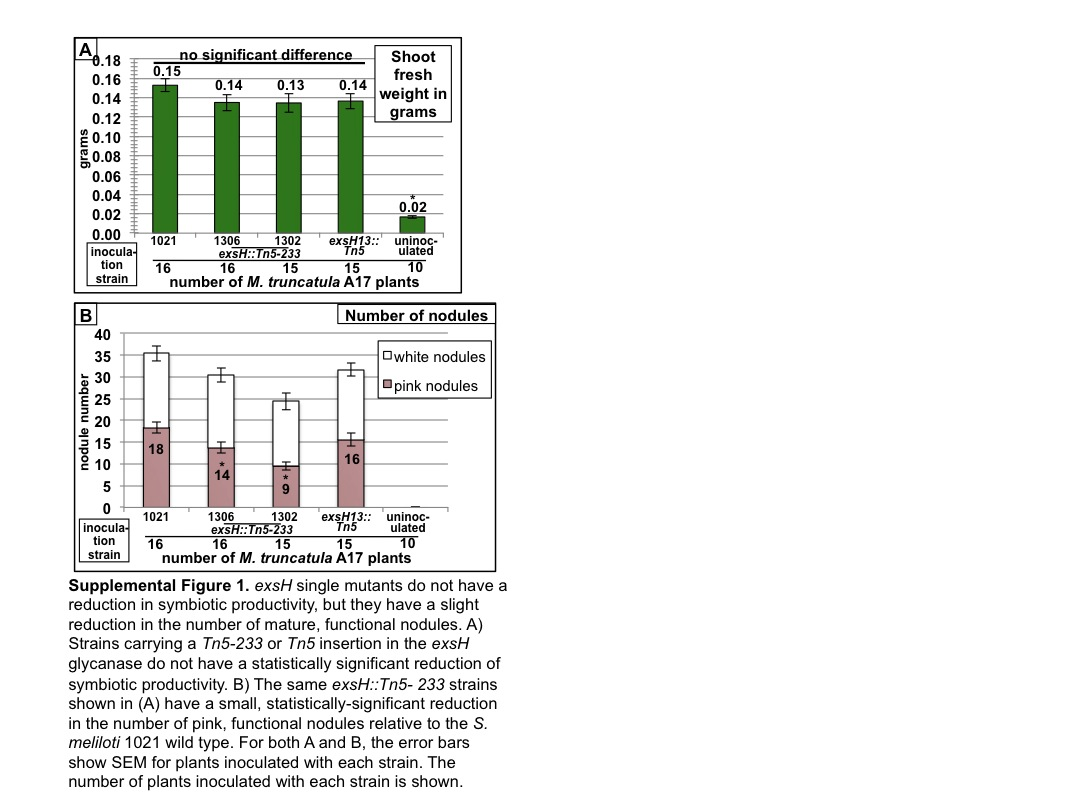

Supplement: Figure S1 — exsH single mutants do not have a reduction in symbiotic productivity, but they have a slight reduction in the number of mature, functional nodules. (A) Strains carrying a Tn5-233 or Tn5 insertion in the exsH glycanase do not have a statistically significant reduction of symbiotic productivity. (B) The same exsH::Tn5-233 strains shown in panel A have a small, statistically significant reduction in the number of pink, functional nodules relative to the S. meliloti 1021 wild type. For both panels A and B, the error bars show SEM for plants inoculated with each strain. The number of plants inoculated with each strain is shown. Download [file mbo003162857sf1.tif]

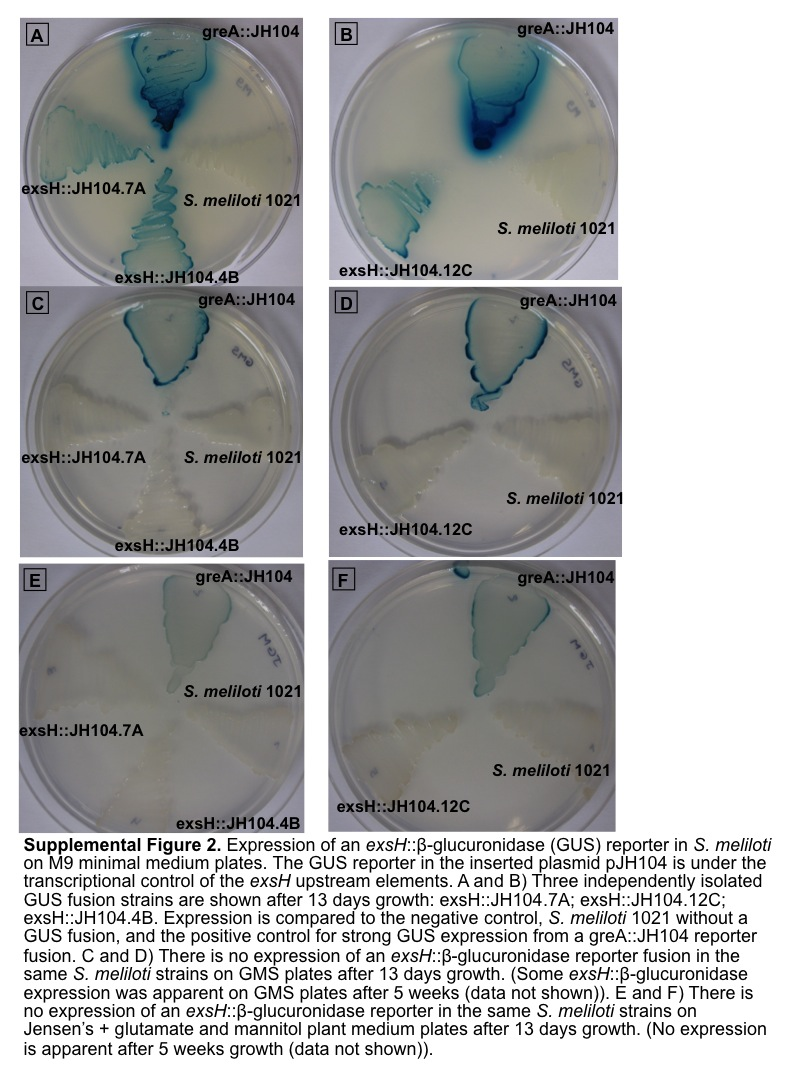

Supplement: Figure S2 — Expression of an exsH::β-glucuronidase (GUS) reporter in S. meliloti on M9 minimal medium plates. The GUS reporter in the inserted plasmid pJH104 is under the transcriptional control of the exsH upstream elements. (A and B) Three independently isolated GUS fusion strains are shown after 13 days of growth: the exsH::JH104.7A, exsH::JH104.12C, and exsH::JH104.4B strains. Expression is compared to that of the negative control, S. meliloti 1021 without a GUS fusion, and the positive control for strong GUS expression from a greA::JH104 reporter fusion. (C and D) There is no expression of an exsH::β-glucuronidase reporter fusion in the same S. meliloti strains on GMS plates after 13 days of growth (Some exsH::β-glucuronidase expression was apparent on GMS plates after 5 weeks [data not shown].) (E and F) There is no expression of an exsH::β-glucuronidase reporter in the same S. meliloti strains on plates containing Jensen’s medium plus glutamate and mannitol plant medium after 13 days of growth. (No expression is apparent after 5 weeks of growth [data not shown].) Download [file mbo003162857sf2.tif]

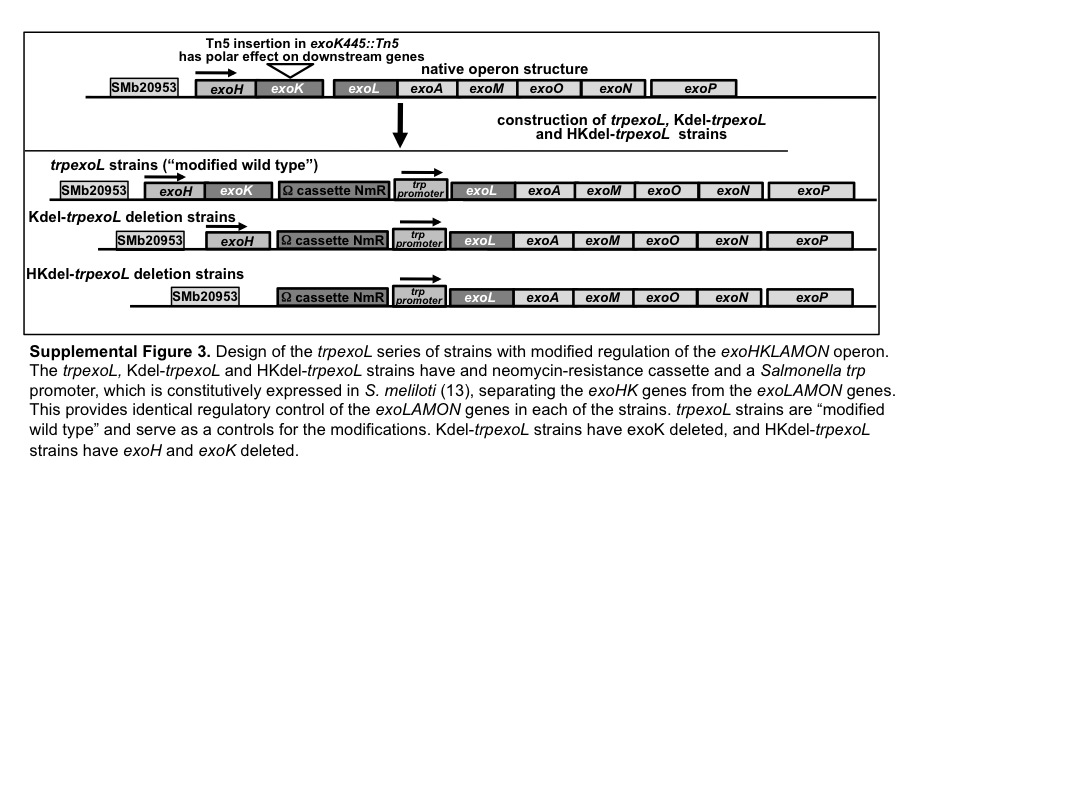

Supplement: Figure S3 — Design of the trpexoL series of strains with modified regulation of the exoHKLAMON operon. The trpexoL, Kdel-trpexoL, and HKdel-trpexoL strains have a neomycin-resistance cassette and a Salmonella trp promoter, which is constitutively expressed in S. meliloti, separating the exoHK genes from the exoLAMON genes. This provides identical regulatory control of the exoLAMON genes in each of the strains. trpexoL strains are “modified wild type” and serve as a controls for the modifications. Kdel-trpexoL strains have exoK deleted, and HKdel-trpexoL strains have exoH and exoK deleted. Download [file mbo003162857sf3.tif]

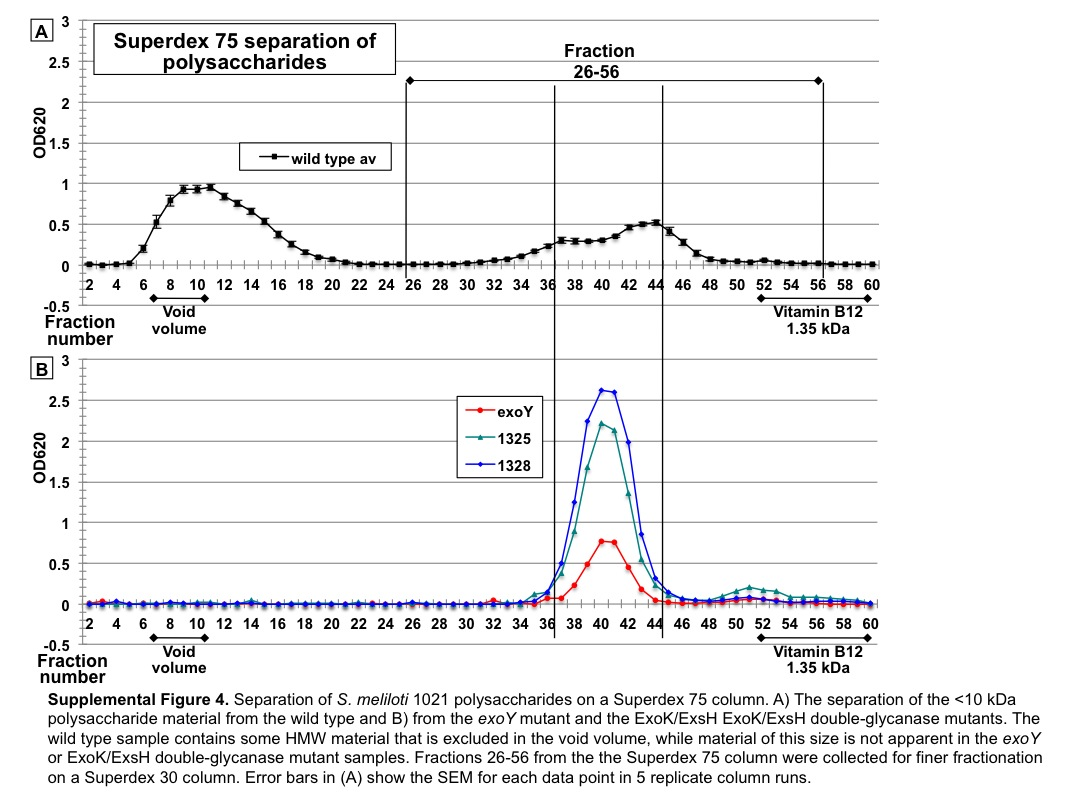

Supplement: Figure S4 — Separation of S. meliloti 1021 polysaccharides on a Superdex 75 column. (A and B) Separation of the <10-kDa polysaccharide material from (A) the wild type and (B) the exoY mutant and the ExoK ExsH ExoK ExsH double glycanase mutants. The wild-type sample contains some HMW material that is excluded in the void volume, while material of this size is not apparent in the exoY or ExoK ExsH double glycanase mutant samples. Fractions 26 to 56 from the Superdex 75 column were collected for finer fractionation on a Superdex 30 column. Error bars in panel A show the SEM for each data point in 5 replicate column runs. Download [file mbo003162857sf4.tif]
