# Supplementary material for: Function of Succinoglycan Polysaccharide in Sinorhizobium meliloti Host Plant Invasion Depends on Succinylation, Not Molecular Weight
Source: mBio. 2016 Jun 21;7(3):e00606-16. doi: 10.1128/mBio.00606-16 (PMC4916376; doi:10.1128/mBio.00606-16)
Supplement: Table S1 — Strains, plasmids, and primers used in this study. [file mbo003162857st1.docx]

**Supplemental Table 1. Strains, plasmids and primers.**

| **Strains** | **Description** | **Reference** |
| --- | --- | --- |
| ***E. coli* strains:** |  |  |
| DH5α M10001) | F- φ80*lac*Z∆M15 ∆(*lac*ZYA-*arg*F) U169 *rec*A1 *end*A1 *hsd*R17 (rk-, mk+) *gal*- *pho*A *sup*E44 λ- *thi*-1 *gyr*A96 *rel*A1 | Life Technologies |
| MM294A M10003) | *pro-82, thi-1, endA, hsdR17, supE44* | ([1](#_ENREF_1)) |
| MT616 M10009) | MM294A *recA56* carrying plasmid pRK600, Cm^R^ | ([2](#_ENREF_2)) |
| ***S. meliloti* 1021 strains** |  |  |
| *S. meliloti* 1021 (KMJ201) | SU47 Sm^R^ | ([3](#_ENREF_3)) |
| *exoY210::Tn5* (KMJ203) | *S. meliloti* 1021 with transposon Tn5 insertion in *exoY*, which encodes the undecaprenyl-phosphate galactose phosphotransferase that catalyzes the first step in succinoglycan biosynthesis | ([4](#_ENREF_4)) |
| *trpexoL*.3Xs3 M961) | *S. meliloti* 1021 with *exoLAMON* operon driven by the *trp* promoter and separated from the *exoHK* genes by an Nm/Km-resistance cassette | ([5](#_ENREF_5)) |
| *trpexoL*.16Xs4 M954) | *S. meliloti* 1021 with *exoLAMON* operon driven by the *trp* promoter and separated from the *exoHK* genes by an Nm/Km-resistance cassette, second independent transductant of *trpexoL*.3Xs3 | ([5](#_ENREF_5)) |
| Kdel-*trpexoL*.1-1 M1014) | *S. meliloti* 1021 with *exoLAMON* operon driven by the *trp* promoter and separated from the *exoH* gene by an Nm/Km-resistance cassette, with *exoK* deletion | ([5](#_ENREF_5)) |
| Kdel-*trpexoL*.2-1 M1015) | *S. meliloti* 1021 with *exoLAMON* operon driven by the *trp* promoter and separated from the *exoH* gene by an Nm/Km-resistance cassette, with *exoK* deletion, 2^nd^ independent isolate | ([5](#_ENREF_5)) |
| Kdel-*trpexoL*.3-3 M1016) | *S. meliloti* 1021 with *exoLAMON* operon driven by the *trp* promoter and separated from the *exoH* gene by an Nm/Km-resistance cassette, with *exoK* deletion, 3^rd^ independent isolate | ([5](#_ENREF_5)) |
| HKdel-*trpexoL*.3-2 (KMJ1006) | *S. meliloti* 1021 with *exoLAMON* operon driven by the *trp* promoter and separated from ORF SMb20953 by an Nm/Km-resistance cassette, with *exoH* and *exoK* deleted, 1^st^ isolate | This study^a^ |
| HKdel-*trpexoL*.2-1 (KMJ1007) | *S. meliloti* 1021 with *exoLAMON* operon driven by the *trp* promoter and separated from ORF SMb20953 by an Nm/Km-resistance cassette, with *exoH* and *exoK* deleted, 2^nd^ independent isolate | This study^a^ |
| HKdel-*trpexoL*.3-2-1 (KMJ1008) | *S. meliloti* 1021 with *exoLAMON* operon driven by the *trp* promoter and separated from ORF SMb20953 by an Nm/Km-resistance cassette, with *exoH* and *exoK* deleted, transductant of KMJ1006 | This study^a^ |
| *exsH13::Tn5* (KMJ219) | *S. meliloti* 1021 with transposon Tn5 insertion in *exsH*, which encodes a succinoglycan glycanase | ([6](#_ENREF_6)) |
| *exsH15::Tn5-233*-1 (KMJ1300) | *S. meliloti* 1021 with transposon Tn5-233 swapped for Tn5 in *exsH*, isolate 1 | This study^b^ |
| *exsH15::Tn5-233*-2 (KMJ1305) | *S. meliloti* 1021 with transposon Tn5-233 swapped for Tn5 in *exsH*, isolate 2 | This study^b^ |
| *exsH14::Tn5-233* transduction strain 1Xs6.3 (KMJ1302) | *S. meliloti* 1021 with transposon Tn5-233 inserted in *exsH*, transduced from strain 1300. | This study^b^ |
| *exsH14::Tn5-233* transduction strain 2Xs6.2 (KMJ1306) | *S. meliloti* 1021 with transposon Tn5-233 inserted in *exsH*, transduced from strain 1305. | This study^b^ |
| SMc00911::JH104 (KMJ855) | *S. meliloti* 1021 carrying an insertion of plasmid pSMc00911::JH104 | ([7](#_ENREF_7)) |
| greA::JH104 (12.4.1a) (KMJ51) | *S. meliloti* 1021 carrying an insertion of plasmid pgreA::JH104 in the *greA* gene | ([7](#_ENREF_7)) |
| *trpexoL//exsH* “modified wild type”/exsH mutant(KMJ1317) | *S. meliloti* 1021 with Tn5-233 transposon insertion from KMJ1305 transduced into KMJ961 | This study^c^ |
| *trpexoL//exsH* “modified wild type”/exsH mutant (KMJ1318) | *S. meliloti* 1021 with Tn5-233 transposon insertion from KMJ1300 transduced into M954 | This study^c^ |
| *trpexoL//exsH* “modified wild type”/exsH mutant (KMJ1319) | *S. meliloti* 1021 with Tn5-233 transposon insertion from KMJ1300 transduced into M954, 2^nd^ independent isolate | This study^c^ |
| Kdel-*trpexoL//exsH* double mutant (KMJ1325), ExoK/ExsH double-glycanase mutant | *S. meliloti* 1021 with Tn5-233 transposon insertion from KMJ1300 transduced into M1014 | This study^c^ |
| Kdel-*trpexoL//exsH* double mutant (KMJ1326), ExoK/ExsH double-glycanase mutant | *S. meliloti* 1021 with Tn5-233 transposon insertion from KMJ1305 transduced into M1014 | This study^c^ |
| Kdel-*trpexoL//exsH* double mutant (KMJ1328), ExoK/ExsH double-glycanase mutant | *S. meliloti* 1021 with Tn5-233 transposon insertion from KMJ1300 transduced into M1015 | This study^c^ |
| Kdel-*trpexoL//exsH* double mutant (KMJ1329), ExoK/ExsH double-glycanase mutant | *S. meliloti* 1021 with Tn5-233 transposon insertion from KMJ1300 transduced into M1015 | This study^c^ |
| Kdel-*trpexoL//exsH* double mutant (KMJ1332), ExoK/ExsH double-glycanase mutant | *S. meliloti* 1021 with Tn5-233 transposon insertion from KMJ1300 transduced into M1016 | This study^c^ |
| Kdel-*trpexoL//exsH* double mutant (KMJ1333), ExoK/ExsH double-glycanase mutant | *S. meliloti* 1021 with Tn5-233 transposon insertion from KMJ1300 transduced into M1016 | This study^c^ |
| HKdel-*trpexoL//exsH* triple mutant (KMJ1342) | *S. meliloti* 1021 with Tn5-233 transposon insertion from KMJ1300 transduced into KMJ1007 | This study^d^ |
| HKdel-*trpexoL//exsH* triple mutant (KMJ1343) | *S. meliloti* 1021 with Tn5-233 transposon insertion from KMJ1305 transduced into KMJ1007 | This study^d^ |
| HKdel-*trpexoL//exsH* triple mutant (KMJ1344) | *S. meliloti* 1021 with Tn5-233 transposon insertion from KMJ1300 transduced into KMJ1006 | This study^d^ |
| HKdel-*trpexoL//exsH* triple mutant (KMJ1345) | *S. meliloti* 1021 with Tn5-233 transposon insertion from KMJ1305 transduced into KMJ1006 | This study^d^ |
| HKdel-*trpexoL//exsH* triple mutant (KMJ1348) | *S. meliloti* 1021 with Tn5-233 transposon insertion from KMJ1300 transduced into KMJ1008 | This study^d^ |
| HKdel-*trpexoL//exsH* triple mutant (KMJ1349) | *S. meliloti* 1021 with Tn5-233 transposon insertion from KMJ1305 transduced into KMJ1008 | This study^d^ |
| exsH::JH104 independently isolated strains 4B (KMJ1360), 7A (KMJ1359), 12C (KMJ1361) | *S. meliloti* 1021 carrying an insertion of plasmid pexsH::JH104, generating a reporter fusion of *exsH* to GUS and a duplication of the 1.4 kb exsH open reading frame. | This study^e^ |
|  |  |  |
| **Plasmids** |  |  |
| pJH104 | GUS-reporter/insertion plasmid (Jeanne Harris, Univ. Vermont) | ([8](#_ENREF_8)) |
| pexsH::JH104 | pJH104 carrying the 1.4 kb exsH open reading frame. | This study |
| pJQ100SK | *sacB* gene-containing suicide vector, Gm^R^ | ([9](#_ENREF_9)) |
| pHP45Ω-Nm/Km | plasmid carrying Nm/Km resistance cassette, flanked by multiple restriction sites | ([10](#_ENREF_10)) |
| ptexoL-ΩKm | intermediate plasmid used in the construction of pK3.trpexo.Km2, Kdelp.texoL, and HKdelp.texoL. Contains Nm/Km-resistance cassette from pHP45Ω-Nm/Km ligated into BamHI site of pJQ100SK and trpexoL fragment ligated into the SalI/XhoI sites of pJQ100SK | ([5](#_ENREF_5)) |
| pK3.trpexo.Km2 | suicide/gene replacement plasmid, places *exoLAMON* under the control of the *trp* promoter, and places Nm/Km-resistance cassette between *exoLAMON* and *exoHK*. Constructed in pJQ100SK | ([5](#_ENREF_5)) |
| Kdelp.texoL | suicide/gene replacement plasmid, places *exoLAMON* under the control of the *trp* promoter, and places Nm/Km-resistance cassette between *exoLAMON* and *exoH*, with simultaneous *exoK* deletion. Constructed in pJQ100SK | ([5](#_ENREF_5)) |
| HKdelp.texoL | suicide/gene replacement plasmid, places *exoLAMON* under the control of the *trp* promoter, and places Nm/Km-resistance cassette between *exoLAMON* and *exoH* upstream region, with simultaneous *exoHK* deletion. Constructed in pJQ100SK | This study^a^ |
|  |  |  |
| **Primers^f^** |  |  |
| trp/exoL.up1.SalI | 5’GCgtcgacAATATACTGAAATAGGTGTTGACATTATTCCATCGA | ([5](#_ENREF_5)) |
| trp/exoL.dwn1 | 5’TCCTGTGCCAGATAGAGTATCTGCAGCATctttgtttcctccCCGCCCGCTATCAGGAAGTGC | ([5](#_ENREF_5)) |
| trp/exoL.up2 | 5’GCACTTCCTGATAGCGGGCGGggaggaaacaaagATGCTGCAGATACTCTATCTGGCACAGGA | ([5](#_ENREF_5)) |
| trp/exoL.dwn2.XhoI | 5’GCctcgagTGCTCCGCACGGACAGGAAA | ([5](#_ENREF_5)) |
| exoK.fwd.SacI | 5’GCgagctcCAATCGATCGCTACAGACGCTTT | ([5](#_ENREF_5)) |
| exoK.rev.XbaI | 5’GCtctagaCTGCAACAAAATGGATAAATTACTCAG | ([5](#_ENREF_5)) |
| Kdel.trpexo1fwd | 5’GCagtactCAGAGGCCTTGCCGATTTCGACT | ([5](#_ENREF_5)) |
| Kdel.trpexo1rev | 5’GCtctagaCGATTGTCATTGTCGTTACCTTTGCTG | ([5](#_ENREF_5)) |
| HKdel.trpexo1fwd | 5’GCagtactGCCGCTTCGTCGGAACGTGT | This study |
| HKdel.trpexo1rev | 5’GCtctagaAGGTTGATGCGCGCCGAGAC | This study |
| exsH.ORF.fwd (primer includes ribosome-binding site) | 5’gcTCTAGAggaggaaacaaagATGAGCAAAACCGTATTGAACGCCGT | This study |
| exsH.ORF.rev | 5’ GCggatccTCAGATCTGCCAGTCCGCGTCG | This study |

^a^Strains HKdel-*trpexoL*.3-2 (KMJ1006), HKdel-*trpexoL*.2-1 (KMJ1007), and HKdel-*trpexoL*.3-2-1 (KMJ1008) were constructed in a similar fashion. Plasmid ptexoL-ΩKm2 ([5](#_ENREF_5)) was digested with Eco53kI and XbaI and ligated with a 1.2-kb XbaI/ScaI fragment that includes the sequence immediately 5’ of *exoH* (amplified with primers HKdel.trpexo1fwd and HKdel.trpexo1rev). This generated plasmid HKdelp.texoL, which was conjugated into *S. meliloti* 1021 to generate strains HKdel-*trpexoL*.3-2 (KMJ1006) and HKdel-*trpexoL*.2-1 (KMJ1007), in which the Nm-resistance cassette has been inserted immediately 5’ of *exoH*, deleting *exoH* and *exoK*, and followed by the *trp* promoter driving expression of the *exoLAMON* operon. Exconjugants were selected on 200 μg/ml neomycin and 1 mg/ml streptomycin. Double-recombinant gene-replacement strains were selected for loss of the *sacB* gene carried by pJQ100SK on 1/10 LBMC + 7% sucrose, with 200 μg/ml neomycin and 1mg/ml streptomycin. The Nm-resistance marker from HKdel-*trpexoL*.3-2 (KMJ1006) was φM12-transduced into *S. meliloti* 1021 to generate strain HKdel-*trpexoL*.3-2-1 (KMJ1008).

^b^The Nm-resistance-carrying *Tn5* transposon inserted in the *exsH* gene in the *exsH15::Tn5* mutant ([6](#_ENREF_6)) was swapped for a gentamicin/spectinomycin-carrying *Tn5-233* transposon using methods previously described ([11](#_ENREF_11)).

^c^We transduced the *exsH::Tn5-233* mutation into the Kdel-*trpexoL exoK* deletion background to generate independently-isolated strains 1325, 1326, 1328, 1329, 1332, and 1333 and into the “modified wild type” background to generate strains 1317, 1318, and 1319. ^d^We transduced the *exsH::Tn5-233* mutation into the HKdel-*trpexoL* deletion background to generate independently-isolated strains 1342, 1343, 1344, 1345, 1348, 1349.

^e^*S. meliloti* 1021 exsH::JH104 GUS-reporters were constructed by amplifying the 1.4 kb *exsH* open reading frame from *S. meliloti* 1021 using primers exsH.ORF.fwd and exsH.ORF.rev and cloning the product into plasmid pJH104 ([8](#_ENREF_8)). The resulting plasmid, pexsH::JH104, was mobilized into *S. meliloti* by triparental conjugation, as described ([2](#_ENREF_2), [11](#_ENREF_11)). Exconjugants were selected on LBMC medium containing 200 μg/ml neomycin and 1 mg/ml streptomycin. The exconjugants exsH::JH104.4B, exsH::JH104.7A, and exsH::JH104.12C were confirmed to have a duplication of the *exsH* open reading frame, generating a GUS reporter fusion to *exsH*.

^f^PCR products were initially cloned into either pCR-Blunt II-TOPO or pCR4-Blunt-TOPO (Life Technologies, Carlsbad, CA).
