# Supplementary material for: Function of Succinoglycan Polysaccharide in Sinorhizobium meliloti Host Plant Invasion Depends on Succinylation, Not Molecular Weight
Source: mBio. 2016 Jun 21;7(3):e00606-16. doi: 10.1128/mBio.00606-16 (PMC4916376; doi:10.1128/mBio.00606-16)
Supplement: Table S2 — Full results of the glycosyl composition analysis. [file mbo003162857st2.docx]

**Supplemental Table 2. Full results of the glycosyl composition analysis.**

| **Sample** | **Strain** | **Superdex 30 column fractions** | **Glucose** | | **Galactose** | | **Arabinose** | | **Rhamnose** | | **Ribose** | | **Fucose** | | **Xylose** | | **Mannose** | | **Total** | |
| --- | --- | --- | --- | --- | --- | --- | --- | --- | --- | --- | --- | --- | --- | --- | --- | --- | --- | --- | --- | --- |
|  |  |  | Mass (μg) | Mol % | Mass (μg) | Mol % | Mass (μg) | Mol % | Mass (μg) | Mol % | Mass (μg) | Mol % | Mass (μg) | Mol % | Mass (μg) | Mol % | Mass (μg) | Mol % | Mass (μg) | Mol % |
| **1** | *S. meliloti* 1021 wild type | 25-28  (peak 2) | 36.4 | 80.0 | 7.5 | 16.6 | n.d. | - | n.d. | - | n.d. | - | n.d. | - | 0.8 | 2.0 | 0.7 | 1.5 | 45.4 | 100 |
| **2** | *S. meliloti* 1021 wild type | 29-34  (peak 3) | 61.4 | 88.6 | 5.5 | 8.0 | 0.3 | .06 | n.d. | - | n.d. | - | n.d. | - | 0.8 | 1.4 | 1.0 | 1.4 | 69.1 | 100 |
| **3** | *S. meliloti* 1021 wild type | 36-41  (peak 4) | 190.6 | 81.3 | 38.3 | 16.4 | 1.3 | 0.7 | n.d. | - | n.d. | - | n.d. | - | 1.7 | 0.9 | 1.8 | 0.8 | 233.8 | 100 |
| **4** | *exoY::Tn5* mutant (no succinoglycan) | 25-28 | 36.4 | 96.5 | 0.2 | .05 | n.d. | - | n.d. | - | n.d. | - | n.d. | - | 0.6 | 1.8 | 0.5 | 1.2 | 37.6 | 100 |
| **5** | *exoY::Tn5* mutant (no succinoglycan) | 29-34 | 5.4 | 94.7 | 0.2 | 3.4 | n.d. | - | n.d. | - | n.d. | - | n.d. | - | n.d. | - | 0.1 | 1.9 | 5.7 | 100 |
| **6** | 1325 double glycanase mutant | 25-28 | 15.5 | 98.4 | 0.1 | 0.7 | n.d. | - | n.d. | - | n.d. | - | n.d. | - | n.d. | - | 0.1 | 0.9 | 15.8 | 100 |
| **7** | 1325 double glycanase mutant | 29-34 | 21.7 | 100.0 | n.d. | - | n.d. | - | n.d. | - | n.d. | - | n.d. | - | n.d. | - | n.d. | - | 21.7 | 100 |
| **8** | 1328 double glycanase mutant | 25-28 | 6.8 | 97.8 | n.d. | - | n.d. | - | n.d. | - | n.d. | - | n.d. | - | n.d. | - | 0.2 | 2.2 | 7.0 | 100 |
| **9** | 1328 double glycanase mutant | 29-34 | 33.2 | 94.9 | 0.6 | 1.6 | n.d. | - | n.d. | - | n.d. | - | n.d. | - | 0.5 | 1.8 | 0.6 | 1.7 | 34.9 | 100 |

n.d. = not detected
